# Unsupervised anomaly detection for longitudinal comparison in whole-body PET/CT images

**DOI:** 10.1007/s11548-026-03672-6

**Published:** 2026-05-25

**Authors:** Takahiro Nakao, Shouhei Hanaoka, Yukihiro Nomura, Takeharu Yoshikawa, Osamu Abe

**Affiliations:** 1 Department of Computational Diagnostic Radiology and Preventive Medicine, The University of Tokyo Hospital, 7-3-1 Hongo, Bunkyo-Ku, Tokyo, Japan; 2 Department of Radiology, The University of Tokyo Hospital, Tokyo, Japan; 3 Division of Radiology and Biomedical Engineering, Graduate School of Medicine, The University of Tokyo, Tokyo, Japan; 4 Center for Frontier Medical Engineering, Chiba University, Chiba, Japan

**Keywords:** FDG-PET, PET/CT, Longitudinal imaging, Deep learning, Unsupervised learning, Anomaly detection

## Abstract

**Purpose:**

This study investigates the utility of unsupervised anomaly detection for longitudinal comparison of whole-body ^18^F-fluorodeoxyglucose (FDG)-PET/CT, which (1) reduces false-positive findings compared with subtraction-based methods and (2) enables highlighting newly appearing lesions across diverse anatomical regions without requiring lesion-annotated datasets, unlike supervised approaches.

**Methods:**

A 3D U-Net was trained exclusively on normal PET/CT images to predict voxel-wise probability distribution parameters of the current PET image conditioned on prior PET, prior CT, and current CT images. During anomaly detection, voxel-wise Z-scores were computed from the predicted parameters and used as an abnormality score map. The performance of the proposed method was compared with that of the subtraction method using slice-wise receiver operating characteristic (ROC) analysis and free-response ROC (FROC) analysis.

**Results:**

The area under the slice-wise ROC curve (AUC) was 0.881 for the proposed method and 0.757 for the subtraction method. Region-wise subanalysis demonstrated AUCs of 0.925, 0.918, and 0.824 in the neck, chest, and abdominal regions, respectively, all of which exceeded the corresponding AUCs obtained with the subtraction method. In the FROC analysis, sensitivity at 5.0 false positives per case was 0.728 for the proposed method, compared with 0.375 for the subtraction method.

**Conclusions:**

The proposed method effectively suppressed false-positive findings compared with subtraction imaging and successfully highlighted lesions across diverse anatomical regions without requiring lesion-annotated datasets, as opposed to supervised learning approaches.

## Introduction

Comparing a current image with prior images from the same patient is a fundamental practice in diagnostic imaging [1]. For example, accurately identifying newly emerging lesions is a critical step toward the early treatment of disease. Nevertheless, comprehensive comparison of whole medical images remains a labor-intensive task, especially for three-dimensional imaging modalities. Therefore, there is a need for systems that support longitudinal image comparison. In this study, we focus on longitudinal comparison and highlighting newly appearing lesions in whole-body ^18^F-fluorodeoxyglucose (FDG)-positron emission tomography/computed tomography (hereafter, PET/CT).

Subtraction has long been a classical approach for automated longitudinal comparison and computer-assisted image interpretation in medical imaging. In practice, it computes the difference between a current image and a registered prior image. This approach has been applied to several tasks, including the detection of new lesions in multiple sclerosis using brain magnetic resonance (MR) images [2, 3] and the detection of bone metastases [4] and lung nodules [5, 6] in CT images. However, such subtraction-based approaches suffer from false positives caused by image misregistration. Misregistration is difficult to eliminate completely in organs that undergo deformation, such as the bowel, even when deformable registration is employed. Moreover, in FDG-PET imaging, strong physiological tracer uptake in the urinary tract can exhibit substantial variability both in shape and intensity across acquisitions. This inevitably leads to false-positive difference signals even in the absence of pathology. Consequently, subtraction is not well suited for longitudinal comparison of whole-body PET/CT.

One approach to reducing such false positives is supervised machine learning. In this paradigm, models are trained to predict the locations of newly appearing lesions using datasets with annotated ground truth. Existing methods include approaches that perform lesion detection or segmentation separately on current and prior images and subsequently integrate the results [7, 8], as well as end-to-end methods that directly segment newly appearing lesions from paired current and prior images [9, 10]. The former strategy has also been reported for whole-body PET/CT [11]. The main drawback of such supervised approaches is that the preparation of annotated training datasets requires considerable time and effort. In addition, although these approaches are effective when targeting specific diseases, they differ fundamentally from subtraction in that they can detect only lesion types the model was trained on. As a result, incidental or unexpected abnormalities may be missed.

Here, we investigate longitudinal comparison based on unsupervised anomaly detection [12, 13]. This framework learns the normal variation observed in a reference (normal) cohort and regards deviations from this variation as anomalies. This approach does not require lesion annotations for training and can capture arbitrary types of abnormalities. Such unsupervised anomaly detection has been well studied in single-time-point images, including PET and other nuclear medicine images [14–16]. For longitudinal analysis, Hoshiai et al. evaluated this approach for computer-aided image interpretation of bone metastases in CT [17]. However, to the best of our knowledge, no studies have yet investigated longitudinal anomaly detection in whole-body PET/CT.

In this study, we investigate the utility of unsupervised anomaly detection for longitudinal comparison of whole-body PET/CT. The contributions of this work are as follows:

1. Compared with the subtraction-based method, our approach reduces false-positive findings.
2. Unlike supervised approaches, it does not require a lesion-annotated dataset and enables highlighting newly appearing lesions across diverse regions of the whole body.

## Materials and methods

### Datasets

#### PET/CT acquisition and image interpretation

The study subjects were adults who visited our hospital for a whole-body medical screening program. As part of this screening program, PET/CT scans were performed using a single scanner (Discovery ST Elite, GE Healthcare, Waukesha, WI). CT images were acquired with the following parameters: field of view (FOV), 500 mm; matrix size, 512 × 512; and voxel size, 0.98 × 0.98 × 1.25 mm. PET images were acquired with the following parameters: FOV, 700 mm; matrix size, 128 × 128; and voxel size, 5.47 × 5.47 × 3.25 mm. PET/CT scans were obtained in three phases (head, early-phase body, and delayed-phase body); however, only early-phase body PET/CT images were included in this study.

All PET/CT images were interpreted using a double-reading approach, in which two radiologists independently reviewed the same PET/CT images, and the final diagnosis was determined by consensus. When non-physiologic FDG uptake was identified, the radiologists determined whether further diagnostic evaluation or treatment at a referral center was required. Hereafter, such FDG uptake is referred to as an *abnormal* finding. Images with any abnormal findings were classified as *abnormal*, whereas those without such findings were classified as *normal*.

#### Case selection and dataset construction

**Fig. 1:**
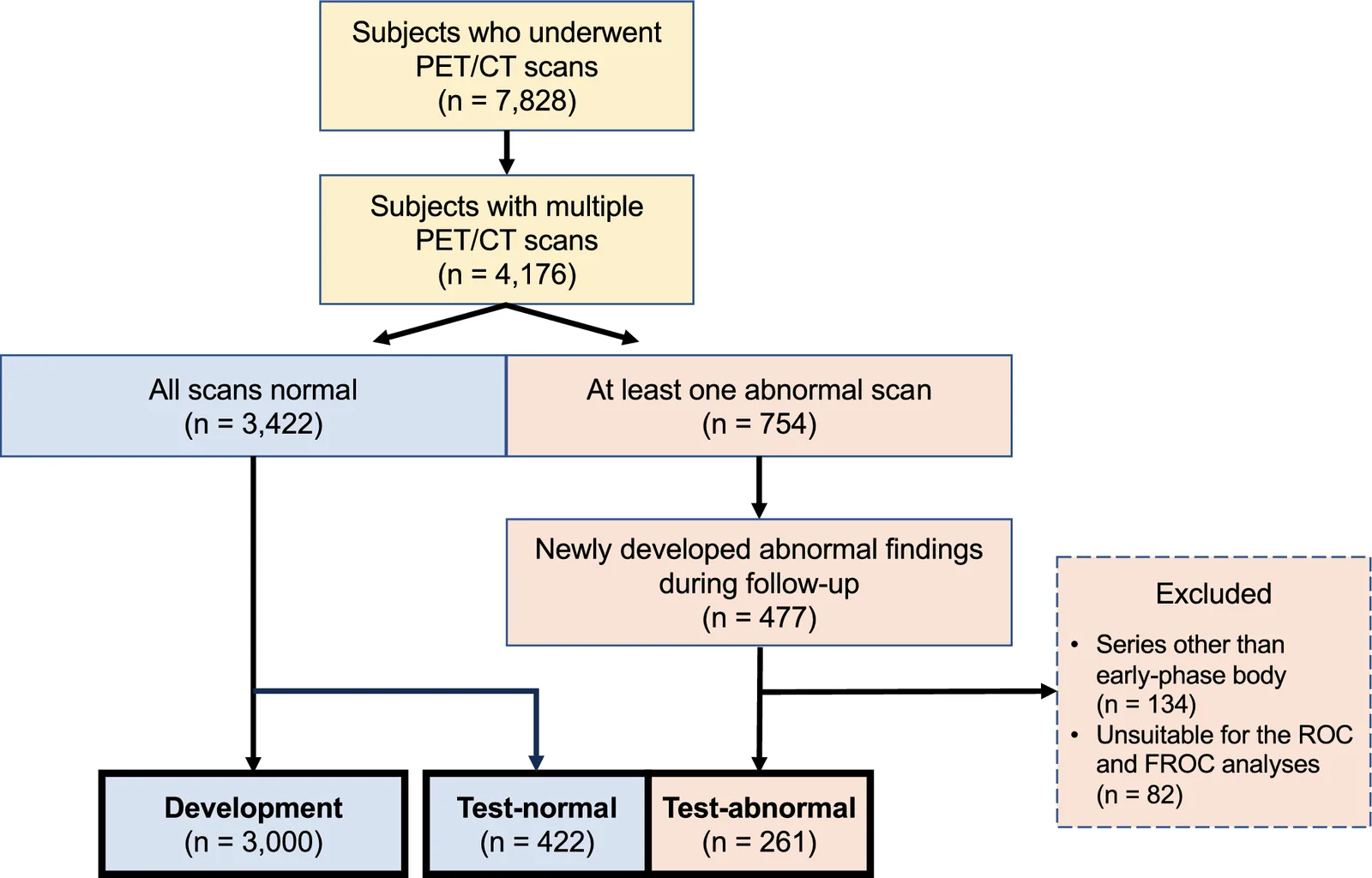
Flowchart of case selection and dataset construction

Figure 1 shows a flowchart of the study inclusion and exclusion process. During the data collection period from October 13, 2006, to November 12, 2017, PET/CT examinations were performed in 7,828 subjects. Among these, 4,176 subjects underwent multiple PET/CT examinations and were included in the present study. PET/CT examinations were typically performed at approximately 1-year intervals.

Among the eligible subjects, 3,422 had only normal PET/CT images throughout the study period according to the double-reading process described above. From these subjects, 3,000 were randomly selected for model development (2,700 for training and 300 for validation). No abnormal images were used during model development.

A total of 754 subjects were diagnosed as having at least one abnormal PET/CT image during the study period. Among them, 477 subjects were newly diagnosed with abnormal findings during follow-up. Cases in which abnormalities were detectable only on image series other than early-phase body PET/CT (i.e., head or delayed-phase body images) were excluded (*n* = 134). The remaining abnormal cases were re-reviewed by two board-certified radiologists, T.N. (10 years of experience) and S.H. (23 years of experience), and 82 cases were further excluded by consensus as unsuitable for the subsequent receiver operating characteristic (ROC) and free-response ROC (FROC) analyses, including cases with non-focal or ill-defined uptake, unclear changes from the prior scan, or insufficient prior-scan information (details are provided in the Appendix). As a result, the remaining 261 abnormal cases were used for performance evaluation. In addition, 422 normal cases that were not used for model development were also included in the performance evaluation.

Table 1 summarizes the size and demographic characteristics of the dataset. The proposed method takes a pair of temporally consecutive scans as input (hereafter referred to as the “prior” and “current” scans). In the test set, only one scan pair was used for each subject. For normal cases, the first two examinations were used as the prior and current scans, respectively. For abnormal cases, the first abnormal examination was used as the current scan, and the immediately preceding normal examination was used as the prior scan. In the development set, all available scans from each subject were used for training; that is, for scans acquired at time points ${t}_{0}, {t}_{1}, {t}_{2}, \cdots$, all possible consecutive pairs ${(t}_{0}, {t}_{1}), ({t}_{1},{t}_{2}), \cdots$, were used.

**Table 1:** Demographic characteristics of the datasets

	No. of Subjects	No. of Scan Pairs	Age	Sex
Development	3,000	10,756	55.2 ± 10.2 (40–86)	M 2029, F 971
Test (normal)	422	422	55.2 ± 10.7 (40–84)	M 274, F 148
Test (abnormal)	261	261	60.9 ± 10.5 (40–91)	M 167, F 94

Age is presented as mean ± standard deviation (range); age corresponds to that at the first visit for the development set and at the time of the prior scan for the test set

### Longitudinal anomaly detection in PET/CT

**Fig. 2:**
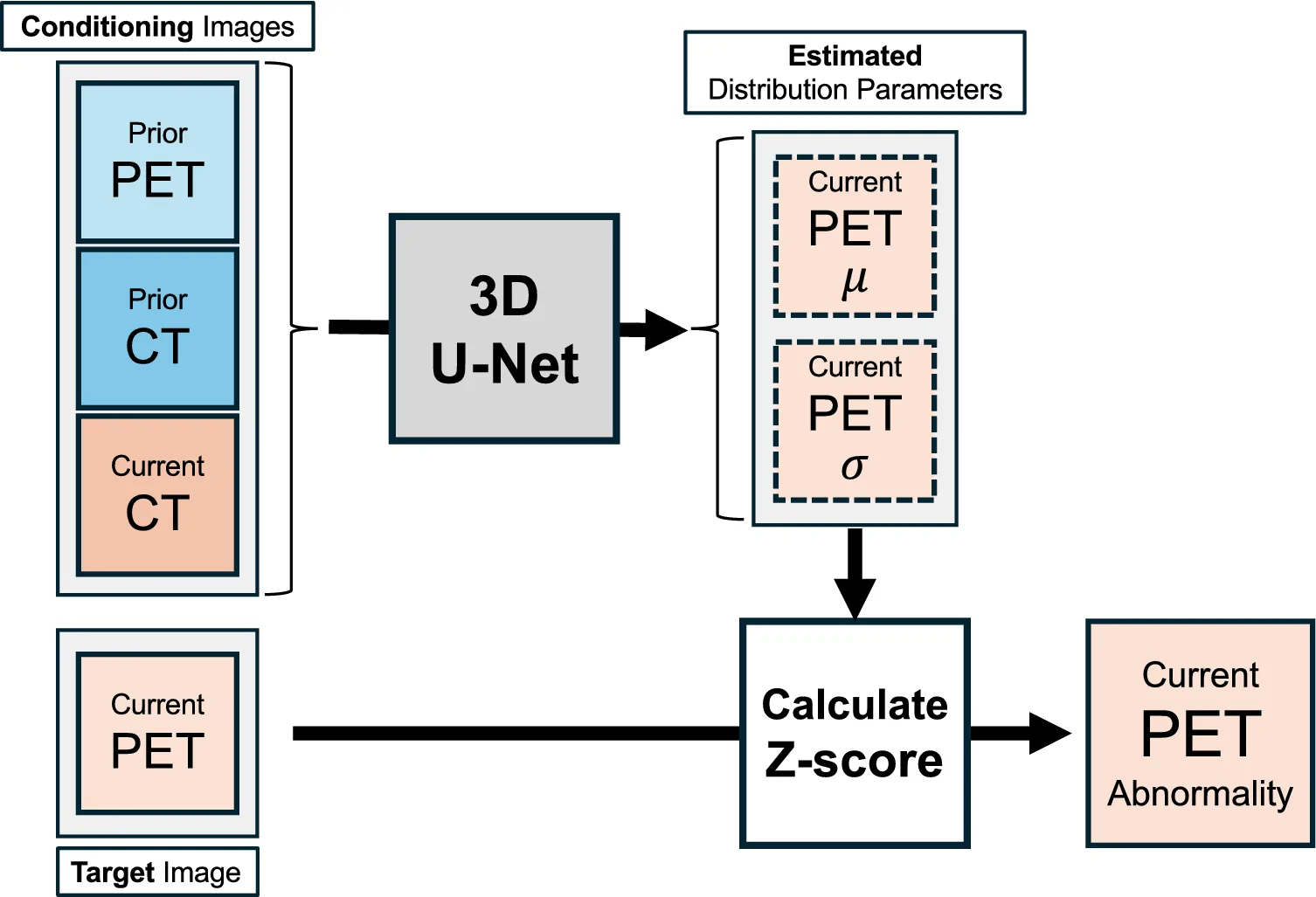
Overview of our longitudinal anomaly detection method. A 3D U-Net predicts the voxel-wise parameters μ and σ of the probability distribution of the current PET image from the conditioning images (prior PET, prior CT, and current CT). Using these predicted parameters and the observed current PET image, Z-scores are computed at each voxel and used as abnormality scores

An overview of the proposed method is shown in Fig. 2. The proposed approach compares prior and current PET/CT images from the same subject and computes an abnormality score for each voxel in the current PET image. Regions with high abnormality scores are more likely to correspond to newly emerged pathological FDG uptake.

First, all CT and PET images were preprocessed by resampling into the same resolution and subsequently co-registering across time points using VoxelMorph [18]; details are provided in the Image Preprocessing section. Next, we trained a neural network with a three-dimensional (3D) U-Net architecture [19, 20], which we refer to as the **distribution parameter estimator**. The network takes the prior PET image as input and predicts the probability distribution of the corresponding current PET image. The estimator is trained only on normal images from the development dataset described above. Intuitively, this model captures the variability between prior and current PET images in normal cases. Furthermore, we incorporated the prior and current CT images as additional inputs to the estimator. Images acquired at different time points, such as prior and current PET images, are susceptible to image misregistration. In contrast, CT and PET images acquired at the same time point (e.g., current CT and current PET) are spatially aligned, as they are obtained sequentially within a single imaging session and do not require additional registration. Therefore, incorporating CT images enables predictions informed by anatomical information from CT and helps mitigate the effects of PET misregistration between prior and current.

Hereafter, we denote the prior PET, current PET, prior CT, and current CT images by $P_{0} , P_{1} , C_{0} ,$ and $C_{1}$, respectively. These are three-dimensional images defined on a voxel grid ${\Omega } \subset {\mathbb{R}}^{3}$. We also define the input to the distribution parameter estimator as the conditioning images ${\mathcal{C}}: = \left( {P_{0} , C_{0} , C_{1} } \right)$. In the proposed method, we assume that the conditional probability distribution of $P_{1}$ given the input ${\mathcal{C}}$ follows a voxel-wise independent Gaussian distribution at each voxel location ${\mathbf{x}} \in {\Omega }$, expressed as

1 $$ \begin{array}{*{20}c} {p_{\theta } (P_{1} | C) =\mathop \prod \limits_{{{\mathbf{x}} \in {\Omega }}} {\mathcal{N}}{(}P_{1} \left( {\mathbf{x}} \right) {|} \mu_{\theta } \left( {{\mathbf{x}};{\mathcal{C}}} \right), \sigma_{\theta } \left( {{\mathbf{x}};{\mathcal{C}}} \right)^{2} ). } \\ \end{array} $$

Here, θ denotes the weights of the distribution parameter estimator. The functions $\mu_{\theta } \left( { \cdot ;{\mathcal{C}}} \right)$ and $\sigma_{\theta } \left( { \cdot ;{\mathcal{C}}} \right)$ represent the mean and standard deviation output by the estimator, respectively, and are three-dimensional images ${\Omega } \to {\mathbb{R}}_{ > 0} { }$.

The distribution parameter estimator is trained via maximum likelihood estimation, which is equivalent to minimizing the negative log-likelihood. Assuming a Gaussian distribution, the loss function used to train the estimator can be written as follows [21]:

2 $$ \begin{array}{*{20}c} {L\left( {\uptheta } \right) = \mathop \sum \limits_{{{\mathrm{x}} \in {\Omega }}} \left( {\frac{{\left( {P_{1} \left( {\mathrm{x}} \right) - \mu_{{\uptheta }} \left( {{\mathrm{x}};{\mathcal{C}}} \right)} \right)^{2} }}{{2\sigma_{{\uptheta }} \left( {{\mathrm{x}};{\mathcal{C}}} \right)^{2} }} + \log \sigma_{{\uptheta }} \left( {{\mathrm{x}};{\mathcal{C}}} \right)} \right) + {\mathrm{const}}{.}} \\ \end{array} $$

During anomaly detection, given the current PET image $P_{1}$ and the outputs of the distribution parameter estimator $\mu_{\theta } \left( {{\textbf{x}};{\mathcal{C}}} \right)$ and $\sigma_{\theta } \left( {{\textbf{x}};{\mathcal{C}}} \right)$, the Z-score $Z\left( {\textbf{x}} \right) \in {\mathbb{R}}$ at each voxel ${\mathbf{x}} \in {\Omega }$ is calculated as

3 $$ \begin{array}{*{20}c} {Z\left( {\mathrm{x}} \right) = \frac{{P_{1} \left( {\mathrm{x}} \right) - \mu_{{\uptheta }} \left( {{\mathrm{x}};{\mathcal{C}}} \right)}}{{\sigma_{{\uptheta }} \left( {{\mathrm{x}};{\mathcal{C}}} \right)}},} \\ \end{array} $$

and used as the voxel-wise abnormality score.

### Image preprocessing

PET voxel values were first converted to standardized uptake values (SUVs) according to the digital imaging and communications in medicine (DICOM) headers. The PET images were then zero-padded to a matrix size of 128 × 128 × 320, using equal padding at both ends of the z-axis. CT images were resampled using linear interpolation to match the resolution and matrix size of the PET images, and their intensity values were normalized to the range of 0 to 1 by applying soft-tissue windowing with a window level of 50 Hounsfield units (HU) and a window width of 400.

We employed VoxelMorph [18], a deep neural network for deformable image registration, for registering prior images to current images. VoxelMorph was trained on the same development set as the distribution parameter estimator. Only CT images were used as inputs to VoxelMorph, and the resulting deformation fields were applied to both CT and PET images. Prior to input into VoxelMorph, CT images were pre-aligned by translation using a phase correlation method.

In addition, body segmentation was performed on CT images using TotalSegmentator 2.10.0 [22]. The resulting body mask was used to restrict loss computation during training and abnormality score calculation during evaluation. Segmentation was performed at the original CT resolution and then resampled to the target resolution using nearest-neighbor interpolation.

### Training details

We employed PyTorch 2.4.1 for deep learning, using the MONAI 1.4.0 implementation of a 3D U-Net for the distribution parameter estimator. The U-Net consisted of five resolution levels with channel sizes of 16, 32, 64, 128, and 256, and downsampling was performed using a stride of 2 at each level. The network took three-channel inputs corresponding to the conditioning images (prior PET, prior CT, and current CT) and produced a two-channel output representing the predicted mean μ and standard deviation σ of the current PET image. All other hyperparameters were left at the MONAI default settings.

For the mean prediction, a softplus activation function was applied to ensure the non-negativity of SUVs. For the standard deviation prediction, the network outputs the logarithm of the standard deviation (log σ) instead of σ for numerical stability. In addition, although log σ can take arbitrary real values, a sigmoid activation function linearly scaled to the range of − 3.0 to 3.0 was used to further stabilize training. The network was optimized using the Adam optimizer [23] with default parameters.

We also used the MONAI 1.4.0 implementation of VoxelMorph, and the network architecture followed that described in the original publication [18]. Cross-correlation was employed as the similarity loss, with the regularization loss weight set to λ=2. Optimization was performed using the Adam optimizer with a learning rate α of ${10}^{-4}$, following the settings of the original study.

All training and inference were performed using two A100 graphics processing units (GPUs) with 40 GB of memory (NVIDIA, Santa Clara, CA, USA). Each model was trained for up to 100 epochs using the training dataset, and the model parameters from the epoch with the minimum loss on the validation dataset were used for the experiments. The batch size was set to the maximum possible given the GPU memory constraints (8 per GPU for the distribution parameter estimator and 5 per GPU for VoxelMorph).

### Performance evaluation

For quantitative performance evaluation, ROC analysis was performed on a slice-wise (axial) basis. The abnormality score for each slice was defined as the maximum Z-score within that slice. All slices from normal cases were used as normal samples, whereas for abnormal cases, only slices containing lesions were used as abnormal samples.

Performance was compared with that of two baseline methods: the PET subtraction image (current minus prior), and the current PET image alone, used as abnormality scores. For these baseline methods, the maximum SUV within each slice was used as the abnormality score for that slice.

In addition, to demonstrate that the proposed method can detect lesions in different anatomical regions, a subanalysis stratified by anatomical region (neck, chest, and abdomen) was conducted. For slices used as normal samples, the slice corresponding to the lung apex was defined as the boundary between the neck and chest, and the slice corresponding to the superior border of the diaphragm was defined as the boundary between the chest and abdomen.

We also conducted an FROC analysis to assess lesion-wise detection performance. Since the proposed method does not directly provide lesion detections or localization results, the anomaly score map was binarized and each connected component was treated as a candidate lesion region. A median filter with a kernel size of 3 was applied as a minimal denoising procedure. The thresholds were set to Z > 2 for the proposed method, SUV > 1 for the subtraction image, and SUV > 2 for the current PET image. A candidate region was considered a true positive if it included the annotated coordinate of a ground-truth lesion, and a false positive otherwise. The maximum anomaly score within each candidate region was used as the confidence score.

Ground-truth labels for the slice-wise ROC and FROC analyses, as well as the anatomical region boundaries used in the subanalysis, were determined by a single board-certified radiologist (T.N.). In the slice-wise ROC analysis, all slices in which newly developed abnormal uptake was visually identifiable were labeled as abnormal. In the FROC analysis, the center point of each newly developed abnormal uptake was defined as the lesion coordinate.

## Results

### Visual examples

**Fig. 3:**
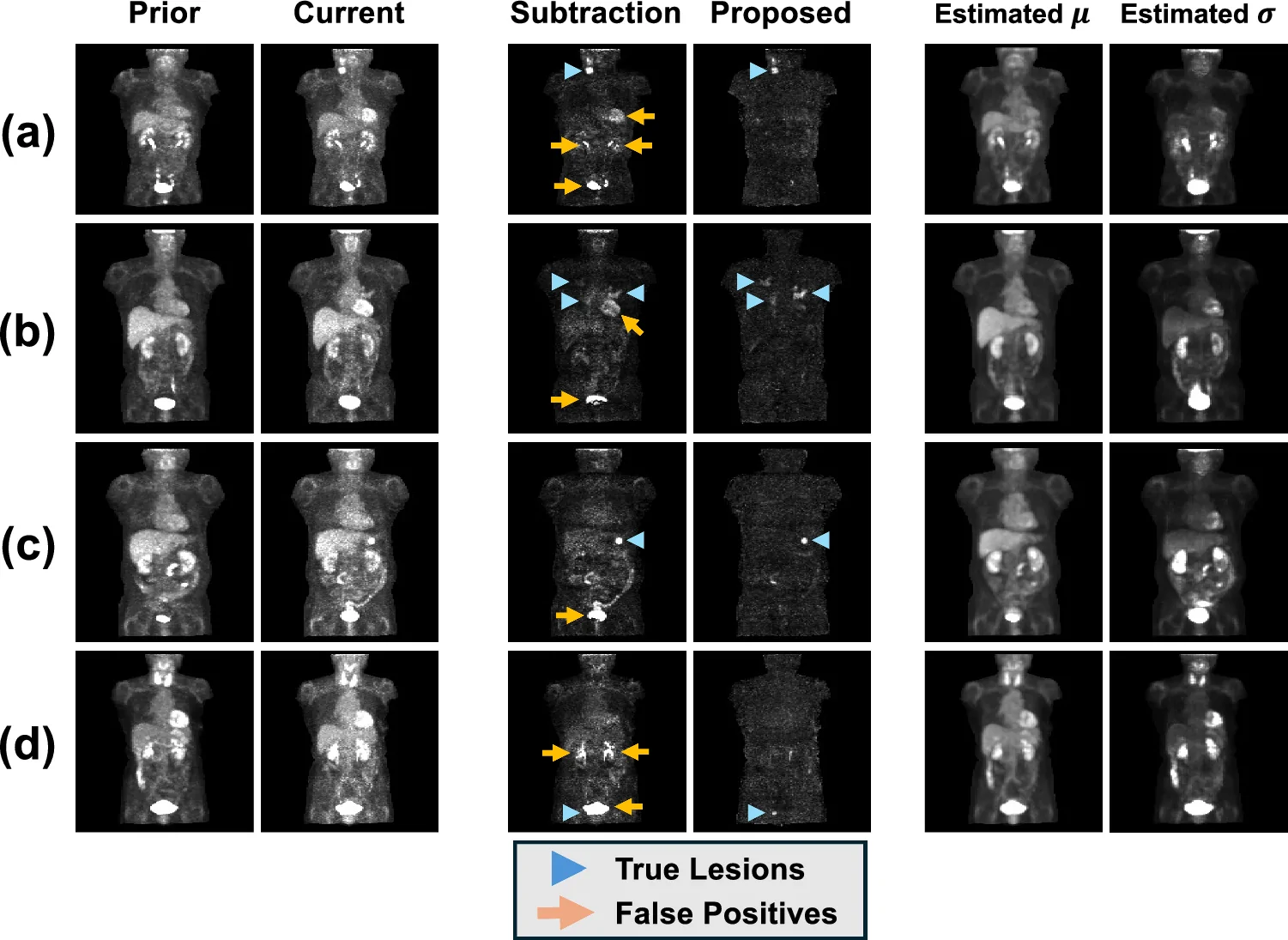
Visual examples of the proposed method. All images are coronal maximum intensity projections (MIPs) of PET images. From left to right, the columns show the prior image, current image, simple subtraction image, Z-score image generated by the proposed method, estimated mean, and estimated standard deviation. Blue arrowheads indicate true lesions, whereas orange arrows indicate false subtraction signals. **a** right submandibular lesion; **b** bilateral lung lesions; **c** gastric lesion; **d** rectal lesion

Examples of the outputs of the proposed method are shown in Fig. 3. In the subtraction images, many false positives appear in regions such as the myocardium and urinary tract; however, the proposed method suppresses most of these false positives and highlights true lesions. The estimated standard deviation σ is high in many of the false-positive regions, indicating high uncertainty in those areas, which contributes to the suppression of these false positives. Especially in Fig. 3d, although the rectal lesion overlaps with the bladder on coronal maximum-intensity projections (MIPs) and is difficult to recognize in the subtraction images, the proposed method clearly visualizes only the true lesion.

### Quantitative performance evaluation

**Fig. 4:**
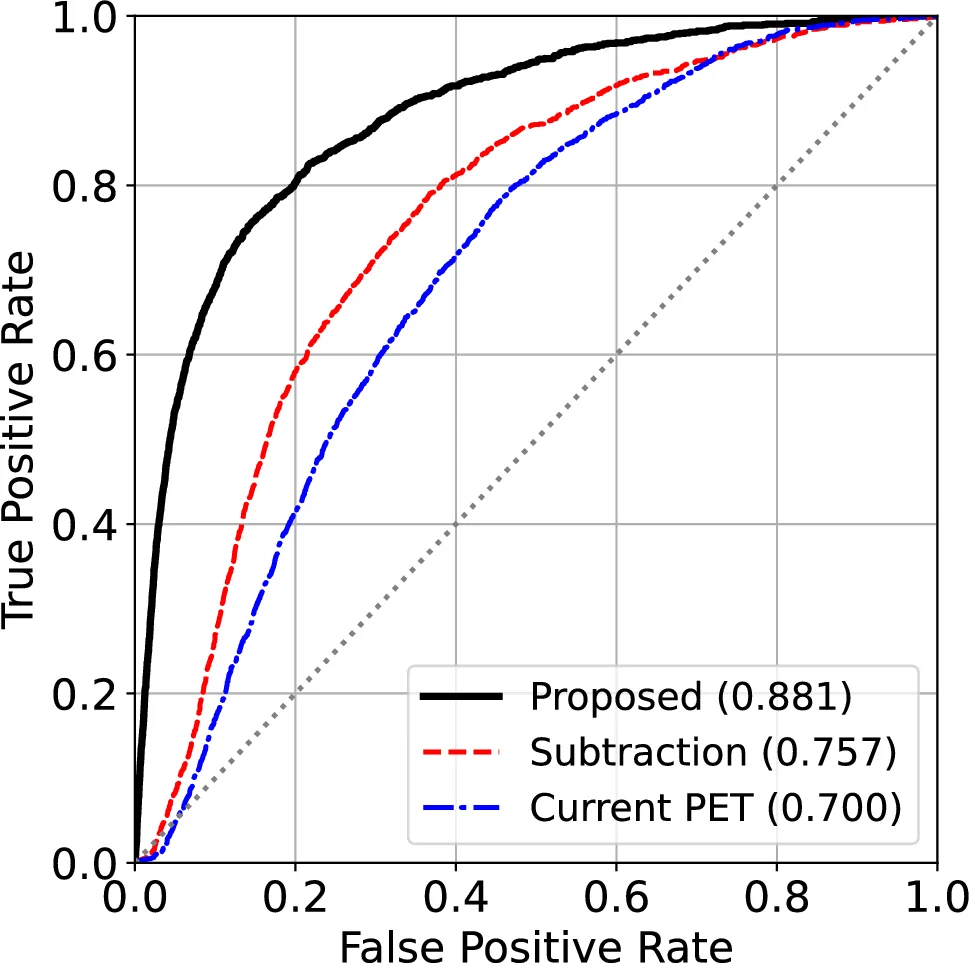
Slice-wise receiver operating characteristic (ROC) curves comparing the proposed method, PET subtraction, and current PET. The numbers in parentheses indicate the area under the curve (AUC)

**Fig. 5:**
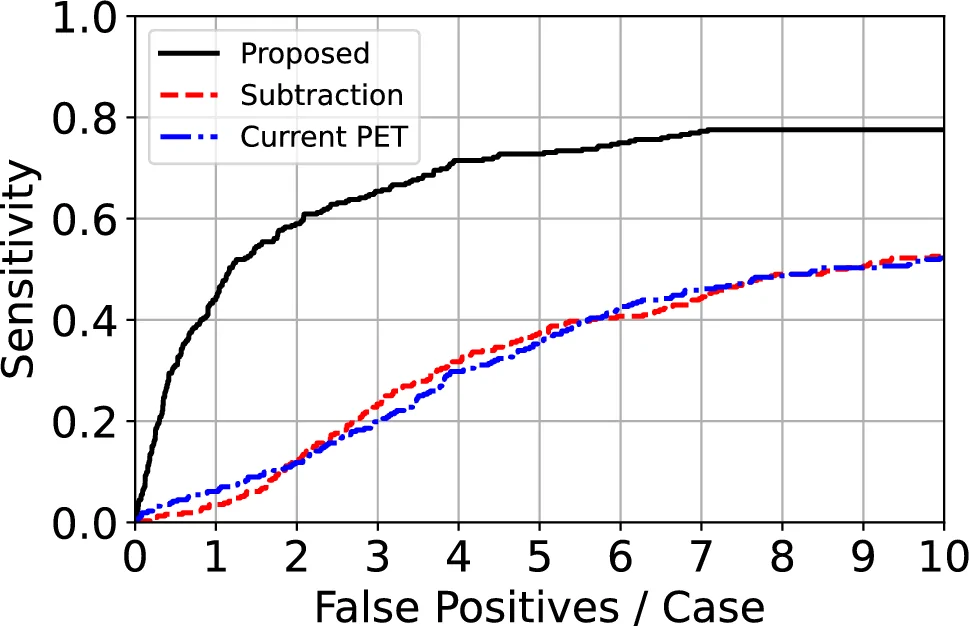
Free-response receiver operating characteristic (FROC) curves comparing the proposed method, PET subtraction, and current PET

The results of the slice-wise ROC analysis are shown in Fig. 4. The area under the ROC curve (AUC) was 0.881 for the proposed method, 0.757 for PET subtraction, and 0.700 for the current PET image. The results of the FROC analysis are shown in Fig. 5. The proposed method showed substantially higher sensitivity than the baseline methods throughout the FROC curve. At 5.0 false positives per case, the sensitivity was 0.728 for the proposed method, compared with 0.375 for PET subtraction and 0.359 for the current PET image. These results indicate that the proposed method has superior anomaly detection performance compared with simple subtraction.

The results of the region-wise subanalysis are presented in Table 2. The proposed method demonstrates robust discriminative performance including in the thoracoabdominal region, where organ deformation and variations in physiological uptake (e.g., myocardium and urinary tract) have a substantial impact.

**Table 2:** Results of the region-wise subanalysis of slice-wise receiver operating characteristic (ROC) analysis

	Proposed	PET Subtraction	Current PET
Neck	0.925	0.881	0.700
Chest	0.918	0.853	0.784
Abdomen	0.824	0.814	0.766
Overall	0.881	0.757	0.700

Values represent the area under the curve (AUC)

### Ablation study

The performance of the proposed method was further compared with two alternative settings: a model using only the prior PET images (*w/o CT*) and a model using only the current CT images (*w/o prior*) as inputs to the distribution parameter estimator. The network architecture was identical to that of the proposed method, except for the number of input channels, and all models were trained under the same conditions. An autoencoder-based unsupervised anomaly detection method using the current PET image alone was also evaluated using the publicly available implementation of Baur et al. [24]. We used the variational autoencoder model provided in the implementation, with all hyperparameters set to their default values.

Table 3 summarizes the results of the ablation study. The proposed method consistently outperformed both the *w/o CT* and *w/o prior* variants, as well as the autoencoder-based method using the current PET image alone, across all anatomical regions. These results indicate that the proposed method effectively uses both prior PET image features and CT-derived anatomical information, leading to more accurate longitudinal anomaly detection.

**Table 3:** Results of the ablation study

	Proposed	w/o Prior	w/o CT	Autoencoder (Current PET) [24]
Neck	**0.925**	0.902	0.892	0.768
Chest	**0.918**	0.913	0.894	0.835
Abdomen	**0.824**	0.805	0.792	0.812
Overall	**0.881**	0.868	0.849	0.750

Values represent the area under the curve (AUC) from slice-wise receiver operating characteristic (ROC) analysis

## Discussion

In this study, we demonstrated the usefulness of unsupervised anomaly detection for longitudinal comparison in whole-body PET/CT images. The proposed method was able to highlight abnormalities across diverse regions and was shown to selectively highlight only truly newly emerged lesions. In addition, unlike supervised machine learning approaches, the proposed method is trained solely on images from normal cases and thus does not require any lesion annotations.

Subtraction is a classical and useful technique for longitudinal comparison in medical imaging; however, it is difficult to completely avoid misregistration, even when deformable registration is applied. In regions with large registration errors, voxel-value variation is also expected to be large. Accordingly, the σ estimated by the proposed method is expected to increase, which in turn leads to lower Z-scores. This likely contributes to reducing false positives caused by misregistration. For example, the shape of the bladder changes from scan to scan, and in the σ map, we observe elevated σ values over a region that encompasses the bladder regions in both the prior and current images. Moreover, particularly in FDG-PET, false-positive differences in subtraction images are unavoidable in regions with strong physiological uptake and/or regions where uptake patterns may vary between scans (e.g., myocardium and urinary tract). By learning the distribution of such variations from a healthy dataset, the proposed method can both emphasize pathological FDG uptake and suppress intensity fluctuations that can also occur in healthy individuals.

To our knowledge, this is the first study to apply unsupervised anomaly detection to the longitudinal comparison of PET/CT images. Unsupervised anomaly detection for medical images has been relatively well studied, including applications to PET/CT [15, 16]. However, its application to longitudinal comparisons has been relatively underexplored. One related study by Hoshiai et al. [17] proposed a deep-learning-based method to reduce false positives in subtraction imaging for the diagnosis of bone metastases on CT; their approach can be viewed as a form of unsupervised anomaly detection similar to ours. However, they did not investigate the superiority of their method over conventional subtraction imaging, and their approach was limited to a single modality, CT. In contrast, our study targets PET/CT, a multimodal examination in which anatomical information from CT acquired simultaneously with PET is used to support interpretation of PET images. In our study, incorporating CT images as inputs to the distribution parameter estimator improved anomaly detection performance compared with using PET images alone. This suggests that anatomical information from CT enables more accurate prediction of the distribution of current PET images.

This study has several limitations. First, the proposed method does not fully automate the detection of new lesions. Instead, it produces an anomaly score map that highlights pathological lesions. In clinical practice, this map would most likely be presented to human readers to support their diagnostic process; however, the actual benefit to readers has yet to be evaluated. Second, the proposed method is primarily designed to highlight newly emerged lesions and is not intended to track changes in existing lesions, such as growth or shrinkage. In studies focusing on change tracking, deformation-field–based approaches are commonly used, and the proposed method could potentially be extended to perform unsupervised anomaly detection on deformation fields obtained through image registration. Third, the proposed method has been validated only on a dataset from a single institution using a single imaging device.

## Conclusion

We demonstrated the usefulness of unsupervised anomaly detection for longitudinal comparison in whole-body PET/CT images. The proposed method does not require lesion annotations, unlike supervised learning approaches, and was shown to suppress false positives caused by variations in physiological uptake, such as in the myocardium and urinary tract, compared with subtraction imaging, while selectively highlighting truly newly emerged lesions.

## Figures and Tables

**Fig. 6:**
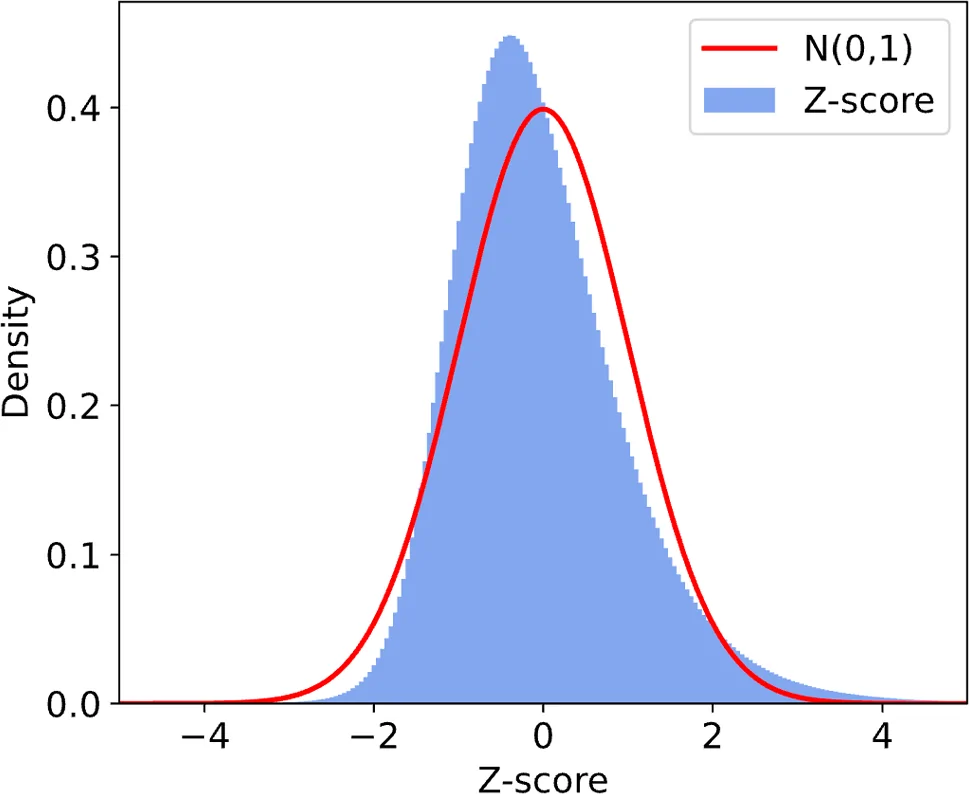
Histogram of Z-scores for the normal cases, overlaid with the standard normal distribution $\mathcal{N}\left(\mathrm{0,1}\right)$

## Appendix

### Dataset Details

See Table 4.

**Table 4:** The detailed breakdown of lesion locations in the test-abnormal set is shown below

Neck		Chest		Abdomen	
Pharynx	11	Lung	146	Liver	4
Larynx	5	Esophagus	5	Pancreas	6
Salivary glands	6	Breast	5	Kidney	2
Thyroid	20	Lymph nodes	10	Adrenal Gland	1
Lymph nodes	12	Bones	3	Prostate	9
Bones	5	Others	2	Uterus	6
Others	2			Stomach	2
				Colon	20
				Small intestine	2
				Appendix	2
				Lymph Nodes	10
				Bones	4
				Others	12

Because a single case may contain multiple lesions, the total count does not match the number of cases in the test-abnormal set. In addition, the images used in this study were acquired for screening purposes, and the final diagnoses are unavailable

### Details of the exclusion criteria for ROC and FROC analyses

All abnormal cases that met the inclusion criteria were re-reviewed by two board-certified radiologists (T.N. and S.H.). Cases considered unsuitable for ROC or free-response ROC (FROC) analyses were excluded according to the criteria as follows:

1. **Non-focal uptake (35 cases):** Cases with diffuse, heterogeneous uptake, such as extensive pneumonia or enteritis, or with multiple closely adjacent lesions that could not be separated, such as clustered enlarged lymph nodes.
2. **Ill-defined boundaries (5 cases):** Cases with diffuse and very faint uptake in which the boundaries of the abnormal region could not be clearly delineated, e.g., subtle interstitial pneumonia.
3. **Pre-existing uptake (10 cases):** Cases in which uptake was already present on the prior scan, making it impossible to clearly identify a specific site on the current scan as a newly emerged abnormality, e.g., worsening of a known chronic lesion such as nontuberculous mycobacterial (NTM) infection.
4. **Unchanged from the prior scan (26 cases):** Cases judged on re-review to show no significant progression compared with the prior scan.
5. **No abnormality (4 cases):** Cases were judged on re-review not to represent pathologic uptake.
6. **Incomplete prior scan coverage (2 cases):** Cases in which the abnormal site identified on the current scan could not be evaluated on the prior scan because it was at the edge of the scan range and was not included on the prior scan (one case), or because the prior examination was incomplete (one case).

### Z-score distribution

Figure ^6^ shows the histogram of Z-scores from the normal cases in the test set. Under the assumption that SUV differences follow a normal distribution, the histogram would follow the standard normal distribution. However, the observed Z-scores show a slightly longer right tail (and a slight leftward shift of the peak to preserve a zero mean). This is likely because SUVs are constrained to be non-negative, making SUV differences more likely to take large positive values. While a non-normal distribution might model SUV differences more accurately, we leave this for future work.

See Fig. 6.
